# PhysVENeT: a physiologically-informed deep learning-based framework for the synthesis of 3D hyperpolarized gas MRI ventilation

**DOI:** 10.1038/s41598-023-38105-w

**Published:** 2023-07-12

**Authors:** Joshua R. Astley, Alberto M. Biancardi, Helen Marshall, Laurie J. Smith, Paul J. C. Hughes, Guilhem J. Collier, Laura C. Saunders, Graham Norquay, Malina-Maria Tofan, Matthew Q. Hatton, Rod Hughes, Jim M. Wild, Bilal A. Tahir

**Affiliations:** 1 Department of Oncology and Metabolism, The University of Sheffield, Sheffield, UK; 2 POLARIS, Department of Infection, Immunity & Cardiovascular Disease, The University of Sheffield, Sheffield, UK; 3 Early Development Respiratory Medicine, AstraZeneca, Cambridge, UK; 4 Insigneo Institute for in Silico Medicine, The University of Sheffield, Sheffield, UK

**Keywords:** Magnetic resonance imaging, Machine learning, Image processing

## Abstract

Functional lung imaging modalities such as hyperpolarized gas MRI ventilation enable visualization and quantification of regional lung ventilation; however, these techniques require specialized equipment and exogenous contrast, limiting clinical adoption. Physiologically-informed techniques to map proton (^1^H)-MRI ventilation have been proposed. These approaches have demonstrated moderate correlation with hyperpolarized gas MRI. Recently, deep learning (DL) has been used for image synthesis applications, including functional lung image synthesis. Here, we propose a 3D multi-channel convolutional neural network that employs physiologically-informed ventilation mapping and multi-inflation structural ^1^H-MRI to synthesize 3D ventilation surrogates (PhysVENeT). The dataset comprised paired inspiratory and expiratory ^1^H-MRI scans and corresponding hyperpolarized gas MRI scans from 170 participants with various pulmonary pathologies. We performed fivefold cross-validation on 150 of these participants and used 20 participants with a previously unseen pathology (post COVID-19) for external validation. Synthetic ventilation surrogates were evaluated using voxel-wise correlation and structural similarity metrics; the proposed PhysVENeT framework significantly outperformed conventional ^1^H-MRI ventilation mapping and other DL approaches which did not utilize structural imaging and ventilation mapping. PhysVENeT can accurately reflect ventilation defects and exhibits minimal overfitting on external validation data compared to DL approaches that do not integrate physiologically-informed mapping.

## Introduction

Pulmonary imaging constitutes a primary component of the clinical workflow of patients with respiratory diseases; various modalities can provide anatomical or functional information that aids in their diagnosis, monitoring, and treatment. Thoracic computed tomography (CT) and proton MRI (^1^H-MRI) are used to ascertain anatomical lung information. However, the relationship between parenchymal destruction and regional function is only somewhat understood. Therefore, functional lung imaging modalities such as single-photon emission CT (SPECT)^1,2^, positron emission tomography (PET)^3,4^ and hyperpolarized gas MRI^5,6^ can be used to glean functional insights. These techniques have shown efficacy in several lung disease applications, including diagnosis, treatment planning and treatment response mapping^7–9^. Hyperpolarized gas MRI is a specialized functional lung imaging modality which has excellent sensitivity to abnormal lung function and allows for the visualization of regional ventilation^10,11^. Hyperpolarized gas MRI can be acquired using either Helium-3 (^3^He) or Xenon-129 (^129^Xe); recently, ^129^Xe has been preferred due to the increased cost and paucity of ^3^He. Tahir et al. demonstrated voxel-wise Spearman’s correlations of ~ 0.8 between ^3^He and ^129^Xe MRI^12^, indicating that there are minimal differences between the two noble gases. Doganay *et* al. compared ^129^Xe-MRI with technetium-99m diethylene-triamine-pentaacetate (^99m^Tc-DTPA) SPECT ventilation imaging^13^, demonstrating a lobar-wise Pearson correlation of 0.64.

To acquire hyperpolarized gas MRI ventilation images, specialized equipment such as a gas polarizer is required, limiting widespread clinical uptake^5^. Surrogates of regional ventilation computed from structural images acquired at different lung inflation levels have been proposed. CT ventilation imaging (CTVI) models regional ventilation from multi-inflation CT, either acquired during tidal breathing (i.e. 4DCT) or at inspiratory and expiratory breath-holds, by assessing changes in regional lung density^14^ or volume^15^. CTVI methods are the subject of intense validation efforts^16^. Tahir et al. demonstrated comparisons between CTVI methods and ^3^He or ^129^Xe MRI, achieving voxel-wise Spearman’s correlations of 0.38 and 0.28, respectively^12^. Kida et al*.* compared CTVI approaches with ^99m^Tc-DTPA SPECT ventilation maps^17^, resulting in voxel-wise Spearman’s correlations between 0.37 and 0.40. Furthermore, the VAMPIRE grand challenge compared various CTVI methods with both Gallium-68 aerosol (Galligas) PET and ^99m^Tc-DTPA SPECT scans^16^, reporting Spearman’s correlations of ~ 0.5. In further investigations, Galligas PET demonstrated a Spearman’s correlation of 0.67 with CTVI maps^18^. In a recent pilot study, structural CT was combined with pulmonary function testing to produce a full-scale airway network (FAN) flow model, generating models of ventilation^19^; these ventilation models were compared to ^129^Xe-MRI and ^99m^Tc-DTPA SPECT ventilation, achieving Spearman’s correlations of 0.67 and 0.68, respectively.

Analogous to CTVI, structural ^1^H-MRI has also been used to derive ^1^H-MRI-based regional ventilation surrogates^20–22^. ^1^H-MRI ventilation maps are derived from differences in signal intensities of co-registered voxels in multi-inflation ^1^H-MRI. The method assumes that these changes reflect naturally occurring density variations in the lungs during breathing^23^. These computational approaches have shown moderate correlation with hyperpolarized gas MRI; Capaldi et al. demonstrated a Spearman’s correlation of 0.67 between ^1^H-MRI- and ^3^He-MRI-derived ventilated lung percentages^24^. Nuclear medicine imaging modalities such as SPECT and PET are ionizing and thus impractical for repeat scanning or scanning of pediatric patients as they require the administration of radioactive tracers such as ^99m^Tc-DTPA and Galligas, respectively, which have several disadvantages, including the presence of clumping artifacts^1,25^. Although hyperpolarized gas MRI is non-ionizing, a contrast agent is still required to produce ventilation images. Unlike CTVI, ^1^H-MRI-based regional ventilation maps can be acquired without contrast and are non-ionizing, enabling its use in pediatric patients and longitudinal applications.

In recent years, deep learning (DL) has been applied to several pulmonary image analysis applications, including image synthesis^26^. Ren et al. used a pre-trained convolutional neural network (CNN) to synthesize SPECT perfusion maps from CT^27^; they employed a dataset comprising 33 lung cancer patients and 137 non-lung cancer patients where the proposed approach generated a voxel-wise Spearman’s correlation of 0.64 averaged across all lobes. Similarly, Liu et al. proposed a CNN-based method to synthesize Technegas SPECT ventilation maps from non-contrast 4DCT using a dataset of 50 participants^28^. They indicate that, after median filtering, the proposed approach achieved a Spearman’s correlation of 0.73 for 10-phase, and 0.71 for 2-phase, 4DCT. Furthermore, Zhong et al.^29^ leveraged a CNN to synthesize CTVI surrogates from 4DCT; they reported a mean ± SD structural similarity index measure (SSIM) of 0.88 ± 0.04^29^. Capaldi et al. used structural free-breathing ^1^H-MRI to synthesize ventilation MRI surrogates for a single 2D coronal section^30^; a 2D UNet CNN with a mean absolute error (MAE) loss function was used. These ventilation surrogates were correlated with ^3^He hyperpolarized gas MRI, achieving a Pearson correlation of 0.87 after six-fold cross-validation on a dataset of 114 participants^30^.

Whilst these approaches have demonstrated the efficacy of CNN-based methods for pulmonary image synthesis, the lack of robustness of these approaches and the inability to produce physiologically consistent results limit clinical applicability. In addition, medical imaging datasets are often limited in size and unrepresentative of a diverse population, limiting the effectiveness of DL techniques. Researchers have proposed the use of hybrid networks which combine computational modeling and DL^31^. Specifically, physics-informed DL frameworks have been used in weather forecasting^32^ and earth surface modeling^33^. Networks integrating computational modeling and DL have also been used for data generation in situations where there is limited data available^34^. Within the medical imaging domain, Poirot et al.^35^ have utilized a physics-based DL approach for dual-energy CT image enhancement.

Here, we propose a physiologically-informed DL framework for the synthesis of fully-volumetric 3D lung ventilation surrogates, leveraging physiologically-based ^1^H-MRI specific ventilation (SV) mapping and structural multi-inflation ^1^H-MRI in a multi-channel CNN configuration. We compare the proposed framework to DL approaches that do not integrate SV mapping or structural ^1^H-MRI and evaluate the quality of synthetic ventilation scans using voxel-wise metrics.

## Materials and methods

### Dataset

The dataset comprised 3D isotropic ^1^H-MRI scans acquired at approximately total lung capacity (TLC) and residual volume (RV), and hyperpolarized ^129^Xe-MRI ventilation scans acquired at functional residual capacity (FRC) + bag (for any given participant, the bag volume was titrated based on standing height with a range of 400 mL–1 L) from 170 healthy participants or patients with various pulmonary pathologies. A summary of participant demographics, stratified by pathology, is provided in Table 1. Imaging data was collected retrospectively from several prospective clinical studies and patients referred for clinical imaging. Data use was approved by the Institutional Review Boards at the University of Sheffield and the National Research Ethics Committee. All data was anonymized and all investigations were conducted in accordance with the relevant guidelines and regulations with participants (or their guardians) providing informed written consent. Appropriate consent and permissions were granted by the Sponsors to utilize this data for retrospective purposes.

**Table 1:** Summary of participant demographic data.

Disease	Number of subjects	Age (years)	Sex	Ventilation defect percentage (%)
		Median (range)	Frequency (%)	Median (range)
Asthma	64	53 (13, 74)	30M (47%), 34F (53%)	2.4 (0.07, 30.9)
Asthma + COPD	23	59 (33, 71)	15M (65%), 8F (35%)	7.0 (1.3, 29.3)
COPD	17	65 (48, 73)	6M (35%), 11F (65%)	18.6 (6.2, 64.8)
Cystic fibrosis	31	18 (9, 48)	16M (52%), 15F (48%)	7.4 (0.42, 56.4)
Healthy	6	38 (26, 71)	3M (50%), 3F (50%)	0.23 (0.03, 0.62)
Investigation for possible airways disease	4	46 (41, 64)	0M (0%), 4F (100%)	6.6 (1.3, 35.0)
Lung cancer	5	73 (68, 79)	4M (80%), 1F (20%)	52.6 (44.9, 69.0)
Post COVID-19	20	58 (25, 73)	18M (90%), 2F (10%)	1.36 (0.55, 5.17)
Total	170	53 (9, 79)	92M (54%), 78F (46%)	3.80 (0.03, 69.0)

### Image acquisition

All participants underwent 3D volumetric ^129^Xe-MRI and ^1^H-MRI in the coronal plane with full lung coverage on a 1.5 T GE HDx scanner (GE Healthcare, Milwaukee, WI, USA). ^1^H-MRI scans were acquired with an 8-element cardiac coil^36^ using a 3D spoiled gradient-recalled sequence with a repetition time/echo time of 1.8/0.7 ms, in-plane resolution of ~ 3 × 3 mm^2^ and a slice thickness of 3 mm. A ~ 35-48 cm field of view with a flip angle of 3° at a bandwidth of 166.6 kHz was used. ^1^H-MRI scans were acquired in approximately 9–12 s at either TLC or RV. Hyperpolarized gas MRI scans were acquired using ^129^Xe that was polarized on site to ~ 25% with an in-house developed rubidium spin-exchange polarizer^37^. A flexible quadrature radiofrequency coil was employed for transmission/reception of MR signals at the Larmor frequency of ^129^Xe-MRI (Clinical MR Solutions, Brookfield, WI, USA). A 3D balanced steady-state free precession sequence was used^36^ with a repetition time/echo time of 6.7/2.2 ms, an in-plane resolution of ~ 4 × 4 mm^2^ and a slice thickness of 10 mm. A ~ 38–40 cm field of view with a flip angle of 9° or 10° and a bandwidth of 16 kHz was used. ^129^Xe-MRI scans were acquired in approximately 10 s at FRC + bag. This results in a total of four scans acquired for each participant, namely, ^1^H-MRI scans acquired at RV and TLC and ^129^Xe-MRI and ^1^H-MRI scans acquired at FRC + bag.

### Image segmentation

To facilitate ^1^H-MRI registration, lung segmentation is required; ^1^H-MRI TLC and RV scans were segmented using a CNN-based generalizable ^1^H-MRI lung segmentation network previously developed by our group^38^. Segmentations were then subsequently manually corrected by several expert observers with the following experience: B.A.T had 10 years, H.M had 7 years, P.J.C.H had 5 years, A.M.B had 5 years and J.R.A had 3 years.

### Image registration

**Figure 1:**
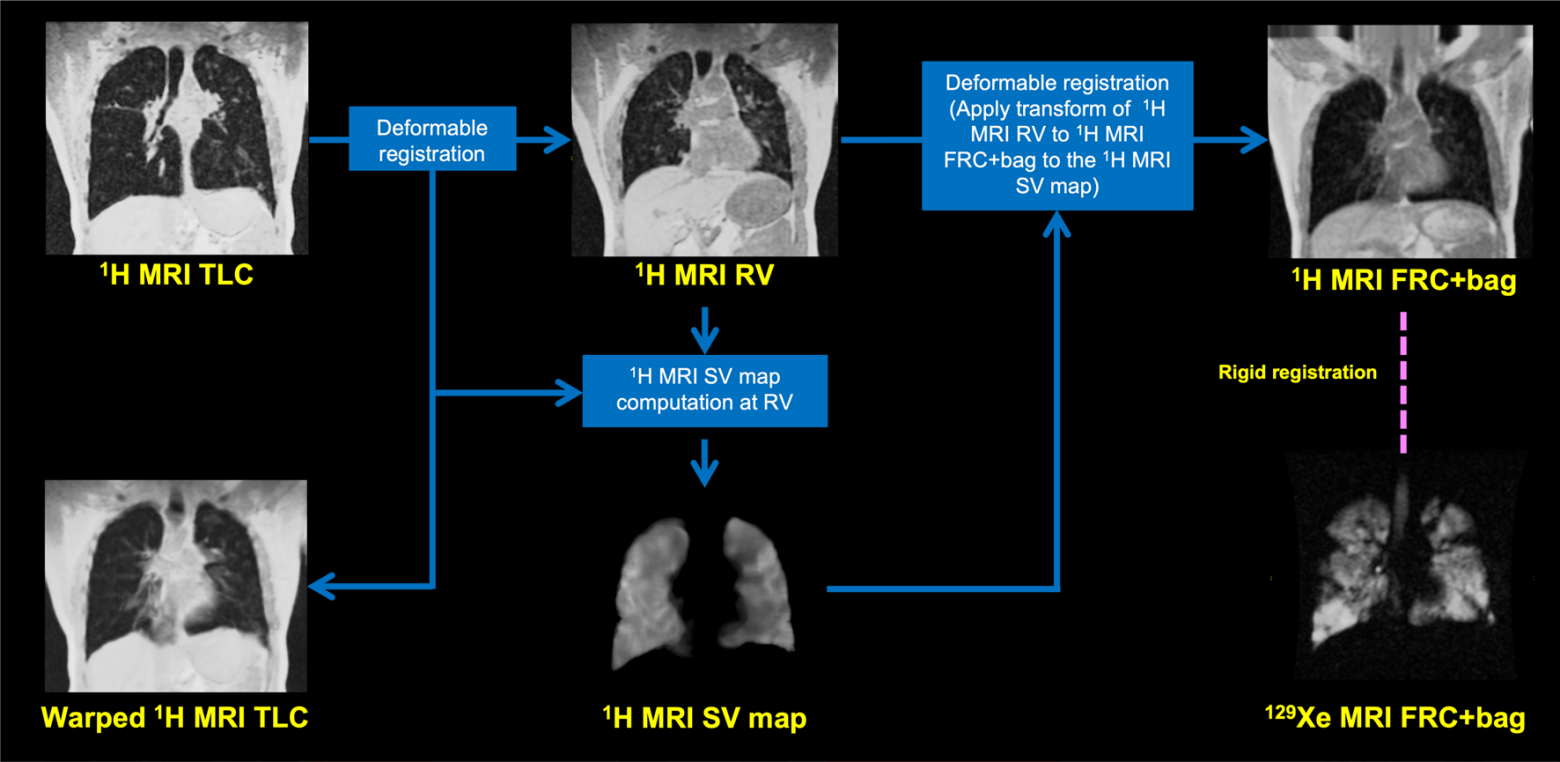
Registration workflow for generating ^1^H-MRI specific ventilation (SV) maps.

RV and TLC ^1^H-MRI scans were aligned using deformable image registration and subsequently registered to the spatial domain and resolution of ^129^Xe-MRI via a corresponding anatomical ^1^H-MRI scan acquired at a similar inflation as ^129^Xe-MRI^12,39^. The registration pipeline consisted of rigid, affine and diffeomorphic steps using the advanced normalization tools (ANTs) registration framework^40^ based on parameters optimized in previous work^41^. The registration pipeline is further described in Fig. 1.

### ^1^H-MRI SV mapping

^1^H-MRI SV maps were computed from the aligned TLC and RV ^1^H-MRI scans. ^1^H-MRI SV maps assume that differences in signal intensities of co-registered voxels in multi-inflation ^1^H-MRI reflect naturally occurring density variations in the lungs during breathing^20,23^. SV is a unitless quantity that aims to model the proportion of inhaled air entering the lungs during normal breathing^24^ and is approximated as follows:

1 $$SV \approx \left(\frac{{SI}_{RV}- {SI}_{TLC}}{{SI}_{TLC}} \right),$$

where *SI*_*RV*_ and *SI*_*TLC*_ are the voxel-wise signal intensities at RV and TLC, respectively. Further details related to the computation of ^1^H-MRI SV maps are included in the Supplementary Material. ^1^H-MRI SV maps were then subsequently median filtered with a radius of 3 × 3 × 1 voxels to account for noise and registration errors.

## Deep learning experiments and evaluation

### CNN architecture configurations

We developed and compared three DL approaches to generate synthetic ^129^Xe-MRI ventilation scans by varying the input images provided to the CNN. These approaches are referred to below:

1. DL (TLC + RV + SV map): *PhysVENeT*.
2. DL (TLC + RV).
3. DL (SV map).

We assessed the effect of providing a physiologically-based ^1^H-MRI SV map, alongside structural TLC and RV ^1^H-MRI scans, as inputs to a CNN (approach 1). This approach, that we call “PhysVENeT”, is compared to a network which is not physiologically-informed (approach 2) and a network which does not integrate structural multi-inflation ^1^H-MRI (approach 3).

**Figure 2:**
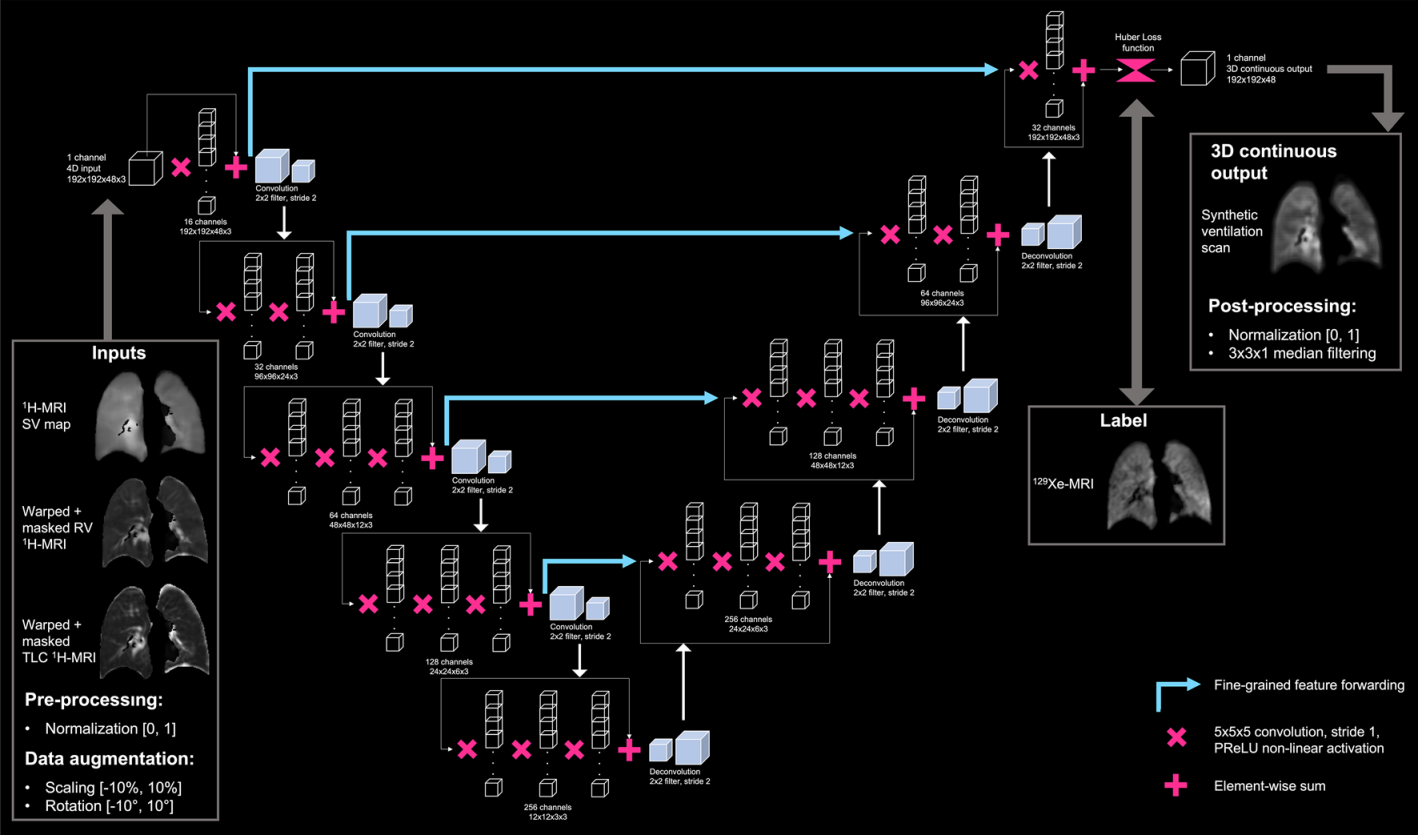
PhysVENeT architecture and training strategy.

For each configuration, input scans with varying dimensions were read by the network using patch-based sampling with patches of 192 × 192 × 48 voxels^42^. The VNet CNN allows for non-isotropic patch sizes in-line with the anisotropic nature of ^129^Xe-MRI. We modified the VNet CNN architecture^43^ to learn functional representations from 3D input scans by outputting a 3D continuous representation of regional ventilation. The CNN contained 16, 32, 64, 128 and 256 feature channels where convolution operations are employed at each layer to both learn residual features and to reduce the resolution of the feature stack, analogous to commonly employed pooling operations. The input layer employs a convolution operation with a 5 × 5 × 5 kernel and stride of 1; two identical convolutions are employed at the second layer and three at the subsequent layers. After each 5 × 5 × 5 convolution, a subsequent 2 × 2 × 2 kernel with stride of 2 was utilized, generating non-overlapping patches; hence, the resolution of the image is divided by two. This is repeated at each layer, resulting in a minimum resolution of 12 × 12 × 3 in the final convolution step. The structure of the network is replicated in deconvolution steps bar the output layer. Each convolution operation employed a PReLU non-linear activation function with valid padding. As indicated by Milletari et al.^43^, the CNN learns residual fine-grained features at each step which informs corresponding deconvolution operations in the upsampling side of the network^43^. The VNet CNN architecture is modified to contain a regression output layer, allowing the network to generate continuous intensity maps in three dimensions. Furthermore, we employ a Huber loss function where the Huber loss (H_Loss_) is defined as:

2 $${H}_{Loss}\left(a\right)= \left\{\begin{array}{l}\frac{1}{2}{a}^{2} for \left|a\right|\le \delta \\ \delta \cdot \left(\left|a\right|-\frac{1}{2}\delta \right) for \left|a\right|> \delta \end{array}\right.,$$

where *a* represents the difference between given co-registered voxels in the ground truth and predicted outputs and δ is defined as 0.1. The Huber loss function is expressed as a representation of either the mean square error (MSE) or the absolute value function at δ. The Huber loss has the benefit of combining the minimum-variance estimator of the MSE loss and the median-unbiased estimator of the absolute value loss to produce a loss function that alternatively provides the sensitivity and robustness of the MSE and absolute loss, respectively. This loss was utilized for synthetic ventilation generation to minimize the impact of outliers in the first stages of training and improve sensitivity once the loss has significantly reduced. For DL approaches 1 and 2, which utilize multiple input images, weight sharing was not employed, resulting in input dimensions of 192 × 192 × 48 × 3 or 192 × 192 × 48 × 2 for the PhysVENeT and other DL configurations, respectively, similar to Kläser et al.^44^ and Jahangir et al.^41^. This method combines the feature maps from spatially aligned TLC and RV ^1^H-MRI alongside the ^1^H-MRI SV map. Therefore, the network can leverage concurrent information distributed across multiple input feature maps^45^. The PhysVENeT architecture (approach 1) is detailed in Fig. 2.

### CNN training parameters

All warped and masked TLC and RV ^1^H-MRI scans and ^129^Xe-MRI ventilation scans underwent pre-processing before they were fed into the network; scans were normalized with image intensities between [0, 1]. Training data was augmented to reduce overfitting whilst still maintaining physiological plausibility. We used an augmentation method where the number of scans in the training set remained consistent; however, each set of input images is deformed using a random rotation and scaling factor between [− 10°, 10°] and [− 10%, 10%], respectively. Different rotation and scaling factors are randomly selected within these limits when the feature map is provided to the network. Thus, the network can be trained for an increased number of epochs as it is highly unlikely to be exposed to the exact same deformations in each epoch. Consequently, we train our network for 900 epochs. Batch normalization was applied at each layer using a mini-batch size of 2 to reduce covariate shift between network layers during training^46^. Network weights were trained from scratch and initialized using Xavier initialization, representing a Gaussian distribution with a mean of 0 and a variance of 1/*N*, where *N* represents the number of weights and biases. The network employs ADAM^47^ optimization with a learning rate of 1 × 10^–5^. L2 regularization and a decay of 1 × 10^–4^ were used to minimize overfitting. The network is trained and tested using the open source medical imaging framework NiftyNet^42^ built on top of TensorFlow 1.1.4^48^. An NVIDIA Tesla V100 GPU with 24 GB of RAM was required for network training. Post-processing was conducted to account for noise and registration errors in synthetic ventilation scans; ^1^H-MRI SV maps and DL-generated synthetic ventilation scans were normalized with signal intensities between [0, 1] and median filtered with a radius of 3 × 3 × 1 voxels.

### Data split

The dataset contained scans from 170 participants. 150 participants were used for fivefold cross-validation, resulting in randomly selected training and testing sets of 120 and 30 participants, respectively, for each fold. The remaining 20 participants were used for external validation; these scans were from participants who had previously been hospitalized for COVID-19 approximately three to six months before imaging, a pathology not contained within the cross-validation dataset. A visual display of the data split, including the cross-validation and external validation procedure is contained in Supplementary Fig. S1.

### Quantitative evaluation

Surrogates of ventilation were quantitatively evaluated using two common voxel-wise image synthesis metrics, namely, the voxel-wise Spearman’s correlation (*rs*) and SSIM. The Spearman’s *rs* was the primary evaluation metric in the CT ventilation imaging grand challenge, VAMPIRE^16^. In a recent review of DL in pulmonary imaging, SSIM was used for evaluation in several image synthesis investigations^26^. Further details of Spearman’s *rs* and SSIM calculations are given in the following sections.

#### Spearman’s correlation

Spearman’s correlation between synthetic ventilation surrogates and corresponding ^129^Xe-MRI scans was assessed at full resolution using Spearman’s *rs*. The correlation was calculated on all voxels within the lung cavity region as defined by the lung volume in a ^1^H-MRI scan acquired at the same inflation as ^129^Xe-MRI. The voxel-wise Spearman’s *rs* quantifies the degree of monotonicity between any two ventilation images within a range of [− 1, 1].

#### SSIM

SSIM is an image quality measure that encompasses similarity information in three domains, namely, the luminance, contrast and structure of the image. SSIM is calculated between non-zero voxels in the reference ^129^Xe-MRI scan (*Xe*) and the synthetic ventilation surrogate (*SVS*) within the lung cavity region, as defined by the lung volume in a ^1^H-MRI scan acquired at the same inflation as ^129^Xe-MRI, as follows:

3 $$SSIM\left(Xe, SVS\right)=\frac{\left(2{\mu }_{Xe}{\mu }_{SVS}+{c}_{1}\right)\left(2{\sigma }_{Xe, SVS}- {c}_{2}\right)}{\left({\mu }_{Xe}^{2}-{\mu }_{SVS}^{2}+{c}_{1}\right)\left({\sigma }_{Xe}^{2}-{\sigma }_{SVS}^{2}+{c}_{2}\right)},$$

where *μ*_*SVS*_ and *μ*_*SVS*_ are the average intensities of *Xe* and *SVS*, respectively, and *σ*_*Xe*_ and *σ*_*SVS*_ are the variances of *Xe* and *SVS*, respectively. *σ*_*Xe,SVS*_ is the covariance of *Xe* and *SVS*. *c*_*1*_ and *c*_*2*_ are defined as follows:

4 $${c}_{1}={\left({k}_{1}L \right)}^{2}, {c}_{2}= {\left({k}_{2}L \right)}^{2},$$

where *L* is the dynamic range of pixel intensities in *Xe* and *SVS* and *k*_*1*_ and *k*_*2*_ are the constants *0.01* and *0.03*, respectively^49^.

### Statistical analysis

We initially determined whether the data was normally distributed via Shapiro–Wilk tests; if normality was not satisfied, non-parametric tests were conducted. Friedman tests with Bonferroni correction for *post-hoc* multiple comparisons were used to assess significances of differences between DL approaches. For each metric, paired *t*-tests were used to assess significances of differences between the DL approaches and the ^1^H-MRI SV map. Wilcoxon tests were used to assess differences between folds on external validation data and differences in performance between the ^1^H-MRI SV map and each fold on the external validation cohort. Statistical analyses were performed using GraphPad Prism 9 (GraphPad, San Diego, CA, USA). In this work, a p-value of < 0.05 was considered statistically significant.

## Results

### Qualitative evaluation

**Figure 3:**
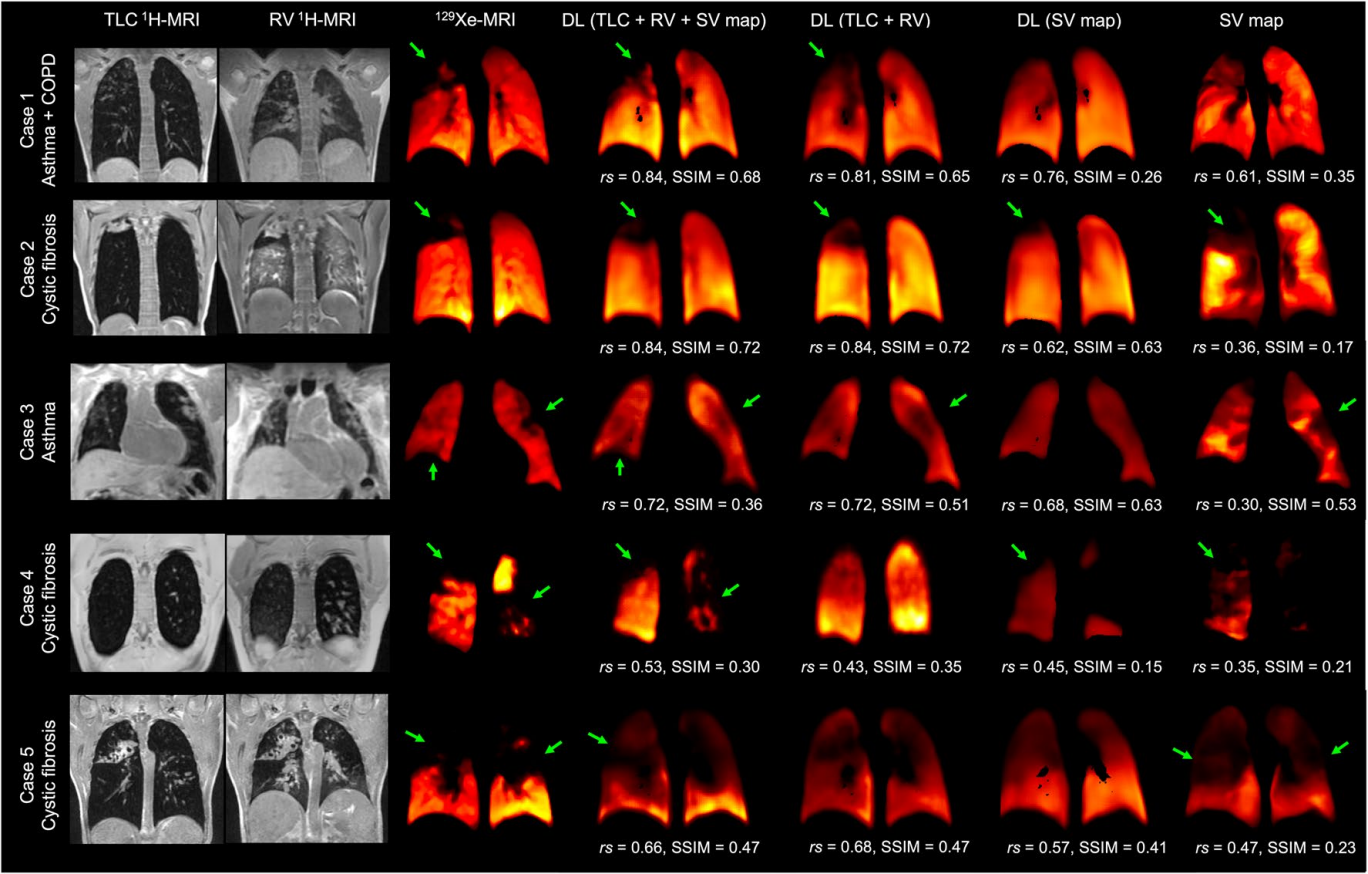
Example coronal slices of TLC and RV ^1^H-MRI, ^129^Xe-MRI, DL-based synthetic ventilation scans and the ^1^H-MRI SV map for five participants in the dataset. Voxel-wise Spearman’s *rs* and SSIM values are given for each DL approach and the ^1^H-MRI SV map. Green arrows indicate ventilation defects in hyperpolarized gas MRI scans which are replicated in synthetic ventilation scans.

Figure 3 shows example coronal slices comparing ^1^H-MRI ventilation surrogates with ^129^Xe-MRI ventilation imaging for five cases within the dataset. Voxel-wise Spearman’s *rs* and SSIM are given for each case and method. Several cases show large ventilation defects which are replicated in synthetic ventilation scans generated by the PhysVENeT framework. Case 3 displays subtle ventilation defects which are somewhat replicated by several synthetic ventilation approaches.

### Quantitative evaluation

**Figure 4:**
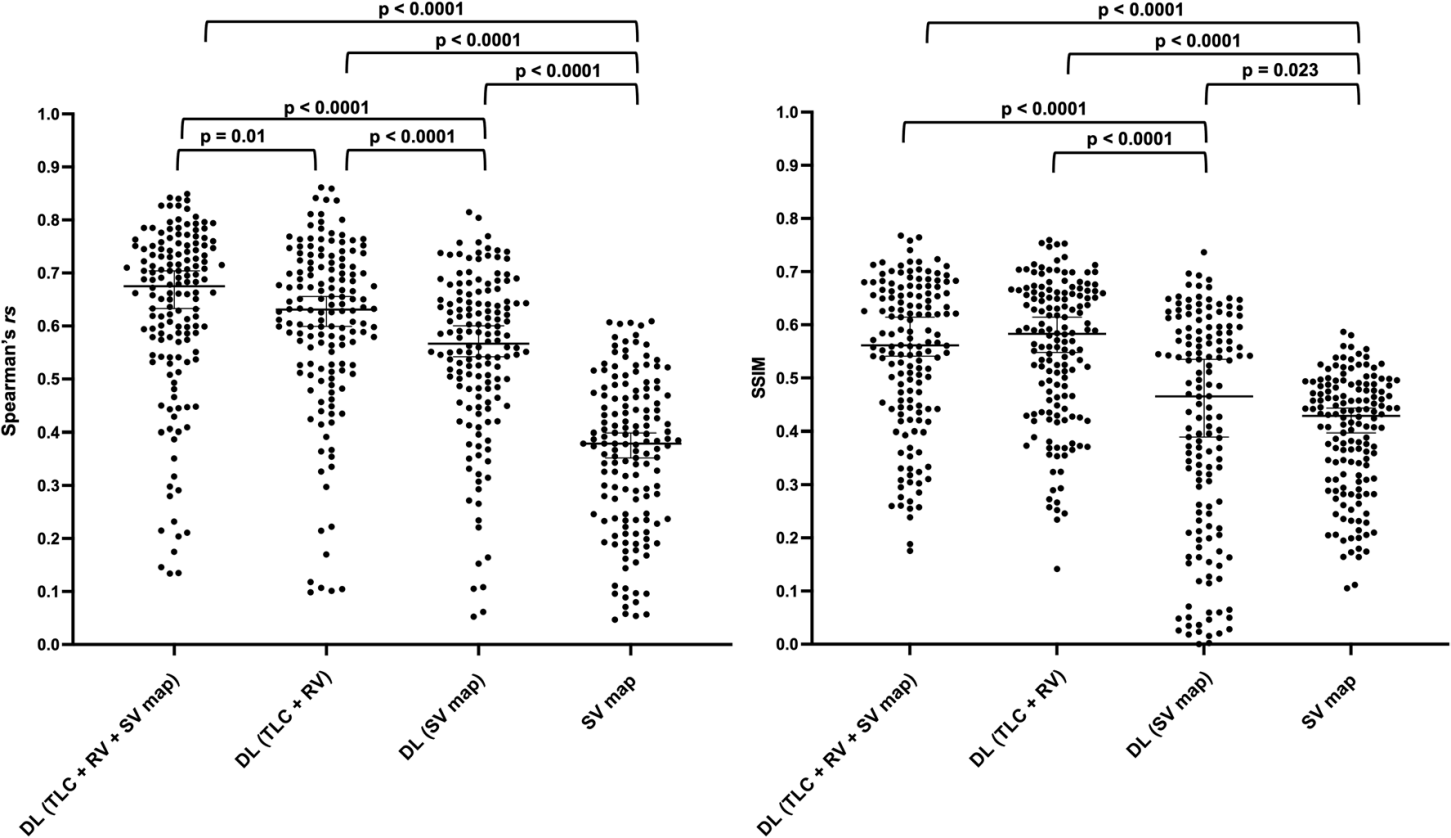
Comparison of performance for DL methods and ^1^H-MRI SV map using the voxel-wise Spearman’s *rs* (left) and SSIM (right) metrics. Significant p-values are provided.

**Figure 5:**
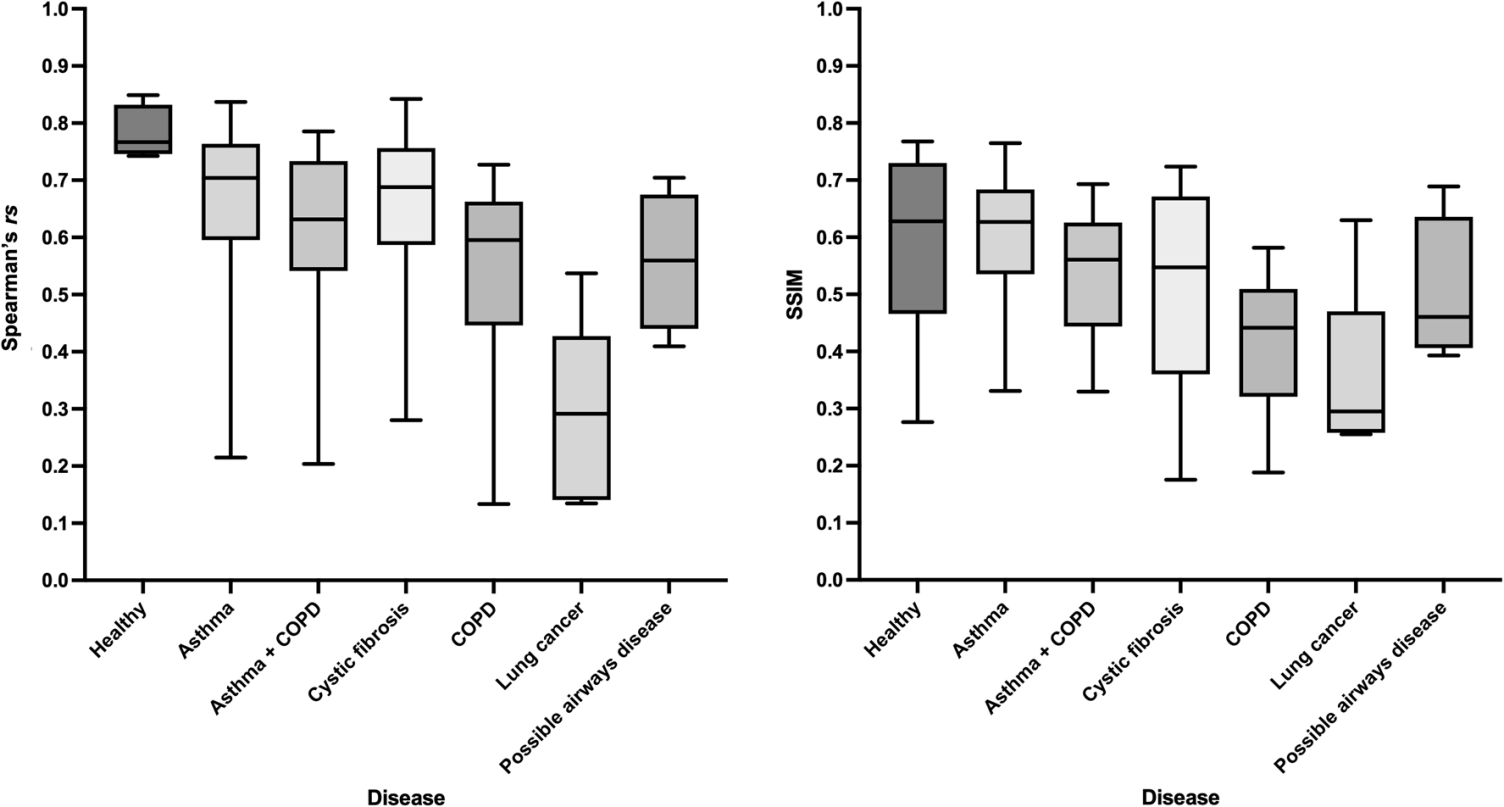
Comparison of performance stratified by participant pathology using Spearman’s *rs* (left) and SSIM (right) metrics for the proposed PhysVENeT framework.

The PhysVENeT framework generated the highest Spearman’s *rs*, achieving a median (range) of 0.68 (0.13, 0.85) and the DL (TLC + RV) approach generated the highest SSIM, achieving a median (range) of 0.58 (0.14, 0.76) when compared to ground-truth ^129^Xe-MRI ventilation. A full summary of results is provided in Table 2. The distribution of Spearman’s *rs* and SSIM for each method across all images within the cross-validation dataset is displayed in Fig. 4; significant p-values are provided. The PhysVENeT significantly outperformed all other DL approaches and ^1^H-MRI SV mapping in terms of Spearman’s *rs*. In addition, both the PhysVENeT and DL (TLC + RV) approaches significantly outperformed the DL (SV map) and ^1^H-MRI SV map using the SSIM metric. No significant difference was observed between the PhysVENeT and DL (TLC + RV) networks using the SSIM metric (p = 0.14). For four participants, the PhysVENeT produced Spearman’s *rs* below 0.2. Figure 5 displays Spearman’s *rs* and SSIM values stratified by disease to assess differences in performance across pathologies for the PhysVENeT. It indicates that the framework generated more accurate synthetic ventilation scans for healthy participants and participants with asthma whilst synthetic ventilation scans were least correlated with ^129^Xe-MRI in lung cancer participants for both metrics used.

**Table 2:** ^1^H-MRI synthetic ventilation results from the SV map and the three DL approaches compared to ^129^Xe-MRI ventilation using the Spearman’s *rs* and SSIM metrics.

Cross-validation	DL (TLC + RV + SV map)		DL (TLC + RV)		DL (SV map)		SV map	
	Spearman’s *rs* Median (range)	SSIM Median (range)	Spearman’s *rs* Median (range)	SSIM Median (range)	Spearman’s *rs* Median (range)	SSIM Median (range)	Spearman’s *rs* Median (range)	SSIM Median (range)
Fold 1	**0.68 (0.13, 0.85)**	0.56 (0.19, 0.77)	0.65 (0.11, 0.86)	**0.57 (0.14, 0.76)**	0.58 (0.06, 0.77)	0.50 (0.05, 0.65)	0.37 (0.09, 0.57)	0.39 (0.11, 0.56)
Fold 2	**0.66 (0.18, 0.84)**	0.54 (0.27, 0.72)	0.60 (0.11, 0.81)	**0.55 (0.27, 0.67)**	0.58 (− 0.04, 0.82)	0.38 (0.01, 0.74)	0.34 (0.05, 0.61)	0.43 (0.17, 0.56)
Fold 3	**0.67 (0.28, 0.79)**	**0.60 (0.29, 0.72)**	0.65 (0.37, 0.80)	0.59 (0.26, 0.75)	0.54 (0.22, 0.69)	0.30 (0.02, 0.64)	0.39 (0.05, 0.61)	0.43 (0.11, 0.59)
Fold 4	**0.69 (0.14, 0.83)**	0.54 (0.19, 0.70)	0.64 (0.10, 0.84)	**0.59 (0.29, 0.71)**	0.54 (0.05, 0.73)	0.55 (0.04, 0.64)	0.41 (− 0.01, 0.60)	0.42 (0.16, 0.52)
Fold 5	**0.66 (0.15, 0.84)**	**0.61 (0.18, 0.76)**	0.63 (0.10, 0.77)	0.59 (0.29, 0.70)	0.64 (0.23, 0.80)	0.54 (0.00, 0.70)	0.38 (0.06, 0.61)	0.45 (0.21, 0.58)
All folds	**0.68 (0.13, 0.85)**	0.56 (0.18, 0.77)	0.63 (0.10, 0.86)	**0.58 (0.14, 0.76)**	0.57 (− 0.04, 0.82)	0.47 (0.00, 0.74)	0.38 (− 0.01, 0.61)	0.43 (0.11, 0.59)

Median (range) is given. Metrics are given for each fold individually and the combined values across all folds.

The best performing approach for each fold is shown in bold.

### External validation

An external validation dataset comprising 20 participants who had a pathology not present in the cross-validation dataset were used to assess the generalizability of DL approaches. The PhysVENeT framework achieved the highest Spearman’s *rs* and SSIM with a median (range) of 0.62 (0.18, 0.79) and 0.58 (0.05, 0.68), respectively, when averaged across all networks trained during each cross-validation fold. The proposed PhysVENeT showed minimal reduction in performance on external validation data, whereas DL approaches that were not physiologically-informed, or did not integrate structural imaging directly, showed larger reductions in both Spearman’s *rs* and SSIM; results for DL approaches are given in Table 3.

**Table 3:** Synthetic ventilation results on the external validation dataset (n = 20) from the three DL approaches compared to ^129^Xe-MRI ventilation using the Spearman’s rs and SSIM metrics.

External validation (n = 20)	DL (TLC + RV + SV map)		DL (TLC + RV)		DL (SV map)	
	Spearman’s *rs* Median (range)	SSIM Median (range)	Spearman’s *rs* Median (range)	SSIM Median (range)	Spearman’s *rs* Median (range)	SSIM Median (range)
Fold 1	0.62 (0.28, 0.76)	**0.58 (0.49, 0.66)**	**0.65 (0.29, 0.82)**	0.56 (0.04, 0.68)	0.53 (0.24, 0.74)	0.53 (0.02, 0.64)
Fold 2	**0.63 (0.23, 0.79)**	**0.57 (0.22, 0.65)**	0.55 (0.24, 0.71)	0.25 (0.01, 0.55)	0.56 (0.41, 0.75)	0.55 (0.03, 0.67)
Fold 3	**0.60 (0.31, 0.77)**	**0.60 (0.05, 0.66)**	0.56 (0.26, 0.73)	0.52 (0.03, 0.63)	0.41 (0.13, 0.64)	0.50 (0.01, 0.56)
Fold 4	**0.61 (0.18, 0.74)**	0.58 (0.33, 0.65)	0.58 (0.21, 0.76)	0.55 (0.04, 0.64)	0.50 (0.27, 0.75)	**0.59 (0.07, 0.66)**
Fold 5	**0.63 (0.22, 0.77)**	**0.58 (0.23, 0.68)**	0.54 (0.18, 0.76)	0.51 (0.05, 0.63)	0.60 (0.26, 0.80)	0.54 (0.03, 0.65)
Average across folds	**0.62 (0.18, 0.79)**	**0.58 (0.05, 0.68)**	0.56 (0.18, 0.82)	0.51 (0.01, 0.68)	0.49 (0.13, 0.80)	0.53 (0.01, 0.66)

Median (range) is given. Metrics are given for ventilation surrogates generated by each of the five folds during cross-validation and the average values across all folds.

The best performing approach for each fold is shown in bold.

**Figure 6:**
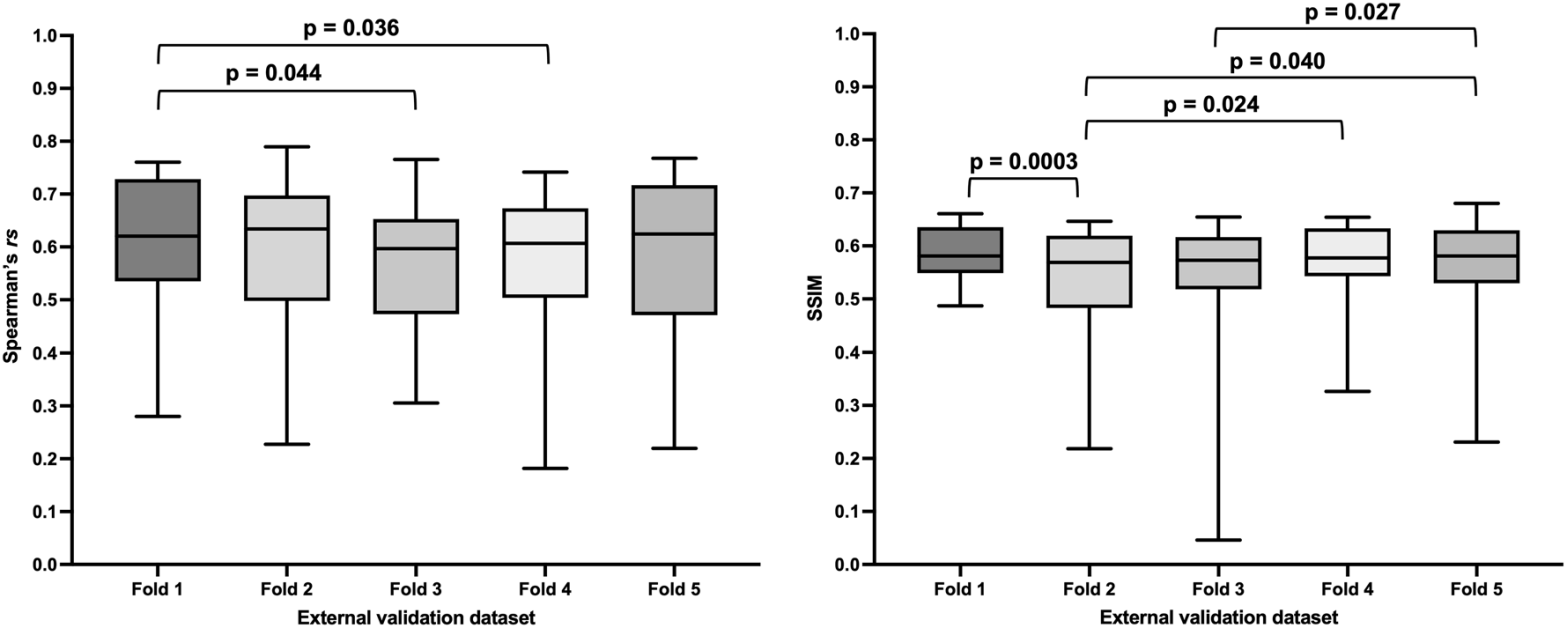
Comparison of performance on external validation data using the five trained models generated by the PhysVENeT during cross-validation in terms of Spearman’s *rs* (left) and SSIM (right). Significant p-values are provided.

Significant differences in performance of the PhysVENeT between networks trained on each cross-validation fold and tested on external validation data were observed; however, the ranges of average Spearman’s *rs* and SSIM values across all folds were narrower than those of other approaches, with a Spearman’s *rs* range of 0.60–0.63 and SSIM range of 0.57–0.60 indicated in Table 3. Significant p-values between the five trained models generated by each fold in the cross-validation process are shown in Fig. 6.

## Discussion

In this work, we propose a framework for the generation of synthetic ventilation surrogates from multi-inflation structural ^1^H-MRI and a physiologically-based SV map. The PhysVENeT approach integrates SV mapping and DL to produce physiologically-informed 3D surrogates of lung ventilation. These synthetic ventilation images correlate with ^129^Xe-MRI ventilation in a voxel-wise manner and can mimic gross ventilation defects across a range of pathologies. Generating 3D synthetic ventilation surrogates from structural imaging modalities, without the requirement of specialized equipment or exogenous contrast, can reduce barriers in the widespread adoption of cutting-edge functional lung imaging modalities, such as hyperpolarized gas MRI.

Synthetic ventilation surrogates generated by the PhysVENeT framework significantly outperformed ^1^H-MRI SV maps. This was demonstrated using the voxel-wise Spearman’s *rs* and SSIM metrics calculated across the whole-lung region where the PhysVENeT achieved a Spearman’s *rs* of 0.68 and an SSIM of 0.56 on the cross-validation dataset. Furthermore, the PhysVENeT significantly outperformed other DL approaches which did not leverage structural ^1^H-MRI or physiologically-based ^1^H-MRI SV mapping, using Spearman’s *rs*. When inference was conducted on external validation data, the PhysVENeT exhibited increased performance compared to other DL approaches, achieving a Spearman’s *rs* of 0.62 and an SSIM of 0.58. The inclusion of both structural ^1^H-MRI and ^1^H-MRI-based SV maps provides PhysVENeT with the ability to generalize effectively to participants of a previously unseen disease. The increase in generalizability on external validation data, in conjunction with significant increases in correlations on cross-validation data, indicates the benefit of using a physiologically-informed framework.

We used a large dataset that contained 170 participants with numerous pulmonary pathologies and varying degrees of lung function, as measured by the ventilation defect percentage (VDP) (Table 1). 150 of these participants were used for five-fold cross-validation, leading to five separately trained networks. The remaining 20 participants were used for external validation whereby each of the five separately trained networks were used to generate ventilation surrogates for these 20 participants. The physiologically-informed PhysVENeT framework performed similarly on both the cross-validation and external validation datasets. In addition, the range of SSIM and Spearman’s *rs* metrics on the external validation data is much narrower than the other DL approaches. Therefore, by leveraging structural ^1^H-MRI and physiologically-informed mapping, the PhysVENeT framework exhibits minimal overfitting and is largely generalizable to scans outside the cross-validation dataset.

The framework uses a VNet CNN backbone previously developed for 3D segmentation tasks^43^. We adapted the VNet with a Huber loss function to output 3D continuous ventilation distributions with the integration of a multi-channel input configuration. The CNN architecture makes use of additional convolution operations to reduce the dimensionality of the image instead of traditional pooling methods. This limits the footprint of the network, reducing the memory consumption^50^. In turn, this facilitates the use of large anisotropic 3D patch sizes. An additional feature of the network architecture is the ability to use anisotropic input dimensions; ^129^Xe-MRI scans have an anisotropic resolution with an in-plane resolution of ~ 4 × 4 mm^2^ and a slice thickness of 10 mm. Thus, we make use of the anisotropic input capabilities of the VNet architecture in contrast to other architectures which require isotropic spatial windowing, such as the nn-UNet^51^.

Previous approaches have utilized DL to generate synthetic ventilation images in 2D. Capaldi et al.^30^ used a 2D UNet CNN with a MAE loss function to generate ventilation images of a single 2D coronal section from free-breathing ^1^H-MRI, limiting volumetric coverage^30^. Moreover, the 2D intensity images cannot contextualize the volumetric nature and spatial clustering of ventilation defects^52^. This can lead to discontinuities between slices which reduces the plausibility of ventilation defect patterns in DL-based ventilation surrogates. Here, we generate fully-volumetric synthetic ventilation surrogates in three dimensions which allows the proposed CNN to learn features which occur over multiple slices.

Levin et al.^53^ has indicated that the resolution of functional lung images need not be higher than the smallest pulmonary gas exchange unit, namely, the acinus. The acinus is approximately 10 × 10 × 10 mm^3^ in adult humans. They also report that the sufficient resolution of ventilation scans can be as low as 20 × 20 × 20 mm^3^ due to the spatial clustering of many ventilation defects^53^. Consequently, we apply 3 × 3 × 1 median filtering as a post-processing step to ^129^Xe-MRI, ^1^H-MRI SV maps, and DL-based synthetic ventilation scans before evaluation. This increases the resolution to 12 × 12 × 10 mm^3^, in-line with appropriate resolutions proposed by Levin et al.^53^.

Contrast-based functional lung imaging modalities such as hyperpolarized gas MRI require specialized equipment and exogenous contrast, which limit their clinical adoption. In addition, functional lung imaging techniques such as CTVI and SPECT expose patients to ionizing radiation and have demonstrated large variability in performance^16^. Furthermore, SPECT has a lower spatial and temporal resolution and a susceptibility to inducing aerosol deposition artifacts when compared to hyperpolarized gas MRI. Therefore, the ability to synthesize hyperpolarized gas MRI ventilation scans in three dimensions from structural non-contrast ^1^H-MRI scans has wide-reaching implications for functional lung imaging, including the potential to be used for functional lung avoidance radiotherapy^7,8^ and treatment response mapping^9^. Kida et al.^17^ has previously demonstrated that a Spearman’s *rs* of ~ 0.4 between CTVI and SPECT images produces clinically indistinguishable radiotherapy plans. Therefore, the reported Spearman’s *rs* in this work of 0.68 between ^129^Xe-MRI and the proposed PhysVENeT indicates its potential clinical utility for functional lung avoidance radiotherapy applications. In addition, ventilation surrogates generated in this work can potentially be used in a triaging capacity for instances where contrast-based functional lung imaging is unavailable.

### Limitations

Despite significant improvements in Spearman’s *rs* and SSIM when compared to ^1^H-MRI SV mapping, the PhysVENeT framework generated only moderate correlations with ^129^Xe-MRI. Synthetic ventilation surrogates were unable to accurately replicate all subtle ventilation defects, and, in some cases, they exhibit minimal correlation. As ^129^Xe-MRI is a direct measure of gas distribution, it can accurately quantify regional ventilation; this characteristic is diminished in synthetic ventilation surrogates where the ability to accurately discern between ventilated and non-ventilated lung regions is reduced. There is a wide range of Spearman’s *rs* values produced by the PhysVENeT framework, ranging between 0.13 and 0.85. Four cases produced Spearman’s *rs* values below 0.2; two of these cases are from lung cancer participants of which there are only five participants in the dataset as a whole, potentially limiting the ability of a DL-based approach to generalize to ventilation distributions exhibited in lung cancer participants. Increasing the number of lung cancer participants in the dataset could improve performance for these cases. This may also be due to the large VDP values present in this cohort, which often lead to increased domain-shift between structural and functional imaging^39^. Additionally, the other two underperforming cases had ^1^H-MRI SV maps that yielded Spearman’s *rs* values below 0.1. The PhysVENeT framework utilizes the ^1^H-MRI SV map as an input and, therefore, if the ^1^H-MRI SV map exhibits poor correlation, it has the potential to impact the performance of the PhysVENeT framework. In future work, it may be appropriate to remove the ^1^H-MRI SV map as an input in cases where its performance is below a certain threshold value.

The repeatability of the proposed approach was not assessed in this work. Nevertheless, the repeatability of ventilation imaging has been previously assessed by our group^54,55^. We employ a robust and standardized protocol for acquiring scans at specific inflation levels. Hughes *et* al. investigated the repeatability of ^3^He hyperpolarized gas MRI ventilation in healthy participants by repeat scanning participants at the lung inflation volumes employed in this study, namely, TLC, RV and FRC + bag^54^. Voxel-wise mean ± SD Spearman’s correlations of 0.93 ± 0.02 for TLC, 0.92 ± 0.03 for RV, and 0.95 ± 0.03 for FRC + bag were achieved; these very high correlation values indicate that there is a high level of repeatability between lung volumes and regional ventilation when using a robust acquisition protocol. In addition, Smith et al. previously demonstrated that, after repeat ^129^Xe-MRI, there was no significant difference in VDP between scans and demonstrated good repeatability with a Bland–Altman bias of 0.2% (LoA =  − 1.4 to 1.8%)^55^. Furthermore, the within-session correlation for VDP was calculated as 0.99, demonstrating the high repeatability of key clinically significant ventilation biomarkers.

In addition, accurate registration is also required for the generation of ventilation surrogates and, therefore, the quality of these registrations significantly impacts the performance of the proposed approach. In future work, an approach independent of registration could be considered. Other DL approaches that utilize generative adversarial networks (GANs) or vision transformers (ViTs) have been used for image synthesis applications^56,57^. The proposed framework used a fully convolutional network that lacks the unsupervised learning benefits of GANs and the long-range feature extraction of ViTs. Future investigations could indicate that utilizing these methods over traditional CNNs leads to improved performance.

The dataset used in this work, whilst varied in pathologies and demographics, is limited in MRI acquisition parameters; all scans were acquired on the same scanner at the same field strength from a single center. Thus, the conclusions of this work cannot be appropriately extended to a dataset of differing sequence or field strength without further investigation. Nevertheless, further expansions of the dataset should focus on the inclusion of a diverse range of MRI acquisition parameters to increase generalizability.

## Conclusion

In this study, we propose a multi-channel CNN to synthesize 3D surrogates of pulmonary ventilation from multi-inflation ^1^H-MRI. These structural scans are combined with an SV map to enhance the physiological plausibility of the synthetic ventilation scans. The PhysVENeT framework produces ventilation surrogates which correlate with ^129^Xe-MRI, reflecting ventilation defects observed in the real scans.

## Supplementary Information

Supplementary Information.

## Data Availability

The imaging datasets generated and/or analyzed during the current study are not publicly available as they were generated as part of an industrial collaborative study that is still underway. Requests for data should be addressed to J.M.W.
