# Supplementary material for: PhysVENeT: a physiologically-informed deep learning-based framework for the synthesis of 3D hyperpolarized gas MRI ventilation
Source: Sci Rep. 2023 Jul 12;13:11273. doi: 10.1038/s41598-023-38105-w (PMC10338553; doi:10.1038/s41598-023-38105-w)
Supplement: Supplementary file 1 — Supplementary Information. [file 41598_2023_38105_MOESM1_ESM.docx]

**PhysVENeT: A physiologically-informed deep learning-based framework for the synthesis of 3D hyperpolarized gas MRI ventilation**

Joshua R. Astley^1,2^, Alberto M Biancardi^2^, Helen Marshall^2^, Laurie J. Smith^2^, Paul J.C. Hughes^2^, Guilhem J. Collier^2^, Laura C. Saunders^2^, Graham Norquay^2^, Malina-Maria Tofan^1^, Matthew Q. Hatton^1^, Rod Hughes^3^, Jim M. Wild^2,4^, Bilal A. Tahir^1,2,4^*

*^1^Department of Oncology and Metabolism, The University of Sheffield, Sheffield, UK*

*^2^POLARIS, Department of Infection, Immunity & Cardiovascular Disease, The University of Sheffield, Sheffield, UK*

*^3^Early Development Respiratory Medicine, AstraZeneca, Cambridge, UK.*

*^4^Insigneo Institute for in silico medicine, The University of Sheffield, Sheffield, UK*

**Supplemental Methods**

**Data split and validation**

The dataset contained scans from 170 participants. 150 participants were used for five-fold cross-validation, resulting in randomly selected training and testing sets of 120 and 30 participants, respectively, for each fold. The remaining 20 participants were used for external validation; these scans were from participants who had previously been hospitalized for COVID-19, a disease not contained within the cross-validation dataset. Supplementary Figure S1 provides a visual representation of the cross-validation and external validation procedure.


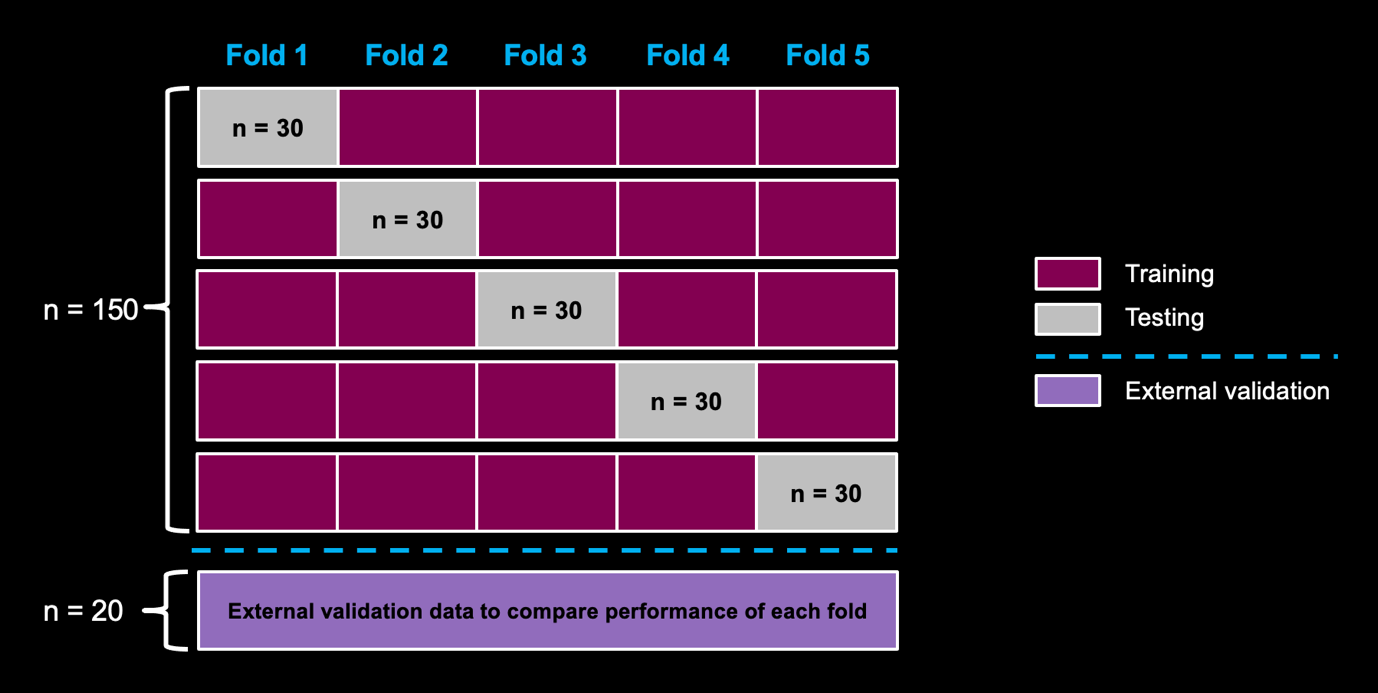
Supplementary Figure S1. Five-fold cross-validation and external validation dataset breakdown.

**^1^H-MRI specific ventilation mapping**

^1^H-MRI specific ventilation (SV) maps were computed from the aligned total lung capacity (TLC) and residual volume (RV) ^1^H-MRI scans. ^1^H-MRI SV mapping assumes that differences in signal intensities of co-registered voxels in multi-inflation ^1^H-MRI reflect naturally occurring density variations in the lungs during breathing[^1^](#_ENREF_1). SV is a unitless quantity that aims to model the proportion of inhaled air entering the lungs during normal breathing[^2^](#_ENREF_2) and is calculated from deformably registered ^1^H-MRI RV and TLC scans as follows:

$$SV=\frac{\Delta V}{{AVF}_{RV}}= \frac{{AVF}_{TLC}- {AVF}_{RV}}{{AVF}_{RV}}$$

( 1 )

where AVF_TLC_ and AVF_RV_ denote the air volume fractions at TLC and RV, respectively. The MRI signal intensity (SI) is known to be approximately inversely proportional to the volume of air in the lung[^3^](#_ENREF_3).

$$SI \tilde{\propto} \frac{1}{AVF}$$

( 2 )

Substituting equation (2) into equation (1) allows the SV to be computed as follows:

$$SV \approx\left( \frac{{SI}_{RV}- {SI}_{TLC}}{{SI}_{TLC}} \right)$$

( 3 )

where *SI_RV_* and *SI_TLC_* are voxel-wise signal intensities at RV and TLC, respectively. ^1^H-MRI SV maps were then subsequently median filtered with a radius of 3x3x1 voxels to account for noise and registration errors.

**References**

1 Kjørstad, Å., Regier, M., Fiehler, J. & Sedlacik, J. A decade of lung expansion: A review of ventilation-weighted 1H lung MRI. *Zeitschrift für Medizinische Physik* **27**, 172-179, doi:<https://doi.org/10.1016/j.zemedi.2016.07.005> (2017).

2 Capaldi, D. P. I. *et al.* Free-breathing Pulmonary MR Imaging to Quantify Regional Ventilation. *Radiology* **287**, 693-704, doi:10.1148/radiol.2018171993 (2018).

3 Zapke, M. *et al.* Magnetic resonance lung function–a breakthrough for lung imaging and functional assessment? A phantom study and clinical trial. *Respir Res* **7**, 1-9 (2006).
